# Preservation of Anti-cytomegalovirus Activity in Human Milk Following High-Pressure Processing Compared to Holder Pasteurization

**DOI:** 10.3389/fnut.2022.918814

**Published:** 2022-05-19

**Authors:** Akash Kothari, Michael A. Pitino, Sharon Unger, Véronique Perreault, Alain Doyen, Yves Pouliot, Allison J. McGeer, Debbie Stone, Deborah L. O’Connor

**Affiliations:** ^1^Translational Medicine Program, Research Institute, The Hospital for Sick Children, Toronto, ON, Canada; ^2^Department of Nutritional Sciences, Temerty Faculty of Medicine, University of Toronto, Toronto, ON, Canada; ^3^Department of Paediatrics, Sinai Health, Toronto, ON, Canada; ^4^Rogers Hixon Ontario Human Milk Bank, Sinai Health, Toronto, ON, Canada; ^5^Centre de Recherche en Sciences et Technologie du Lait (STELA), Département des Sciences des Aliments et de Nutrition, Institut sur la Nutrition et les Aliments Fonctionnels, Université Laval, Quebec City, QC, Canada; ^6^Institute of Health Policy, Management and Evaluation, Dalla Lana School of Public Health, University of Toronto, Toronto, ON, Canada; ^7^Laboratory Medicine and Pathobiology, Temerty Faculty of Medicine, University of Toronto, Toronto, ON, Canada; ^8^Department of Microbiology, Sinai Health, Toronto, ON, Canada

**Keywords:** Holder pasteurization, high-pressure processing (HPP), cytomegalovirus (CMV), hepatitis A virus (HAV), antiviral, donor human milk

## Abstract

Pasteurized donor human milk is recommended for hospitalized preterm infants when mother’s own milk is unavailable. Our aim was to compare the antiviral activity of human milk processed by Holder pasteurization (HoP) or high-pressure processing (HPP) against representative enveloped and non-enveloped viruses including cytomegalovirus and hepatitis A virus. Expressed milk from 20 donors collected from the Ontario Milk Bank was combined into 10 pools, each from two unique donors. Each pool was processed by HoP (62.5°C, 30 min) or HPP (500 MPa, 8 min, 4°C) and subsequently inoculated with cytomegalovirus or hepatitis A virus to achieve a final concentration of 5-log plaque-forming units/mL. Plaque reduction assays were used to quantify detectable virus after 30 min incubation (room temperature). *Post hoc* experiments using a 4 h incubation time were conducted if reductions were detected at 30 min. Irrespective of processing, cytomegalovirus concentrations declined in all pools after 30 min incubation (*P* < 0.0001). Milk processed by HoP exhibited significantly less reduction compared to raw milk (*P* = 0.0069). In *post hoc* experiments, anti-cytomegalovirus activity was maintained at 4 h, with high inter-pool variability. Hepatitis A virus concentration remained unchanged after 30 min incubation in raw and processed milk. Anti-cytomegalovirus activity in human milk is preserved following HoP and HPP, persisting up to 4 h post-inoculation; anti-hepatitis A virus activity was not observed in raw or processed milk. Further research is needed to understand how HoP or promising alternative processing methods affect the antiviral activity of donated milk, given its potential importance to recipient infants.

## Introduction

Mother’s own milk provides infants with many immunological and bioactive proteins that protect against infection (1, 2). When an adequate volume of mother’s own milk is unavailable, supplemental pasteurized donor human milk is preferred for hospitalized preterm infants, over formula, as it reduces their risk for necrotizing enterocolitis (3, 4). Donor human milk in North America undergoes Holder pasteurization (HoP) (62.5°C, 30 min), a heat treatment sufficient to inactivate pathogens, including viruses, that could harm an infant (5, 6). Many of the immunological and bioactive components in human milk that are antiviral, including lactoferrin, lysozyme, and some immunoglobulins (Ig), are heat-sensitive and are negatively impacted by HoP (7–9). Independent of processing, certain donor characteristics including pre-pregnancy body mass index, preterm birth, and postpartum day of milk expression may also impact the concentrations of these components and resulting antiviral activity (10–12).

The antiviral capacity of human milk is well described in the literature. For example, human milk inoculated with concentrated Ebola virus or severe acute respiratory syndrome coronavirus-2 (SARS-CoV-2) reduced levels of infectious virus by >1-log (13, 14). Human cytomegalovirus (CMV), an enveloped virus that can be secreted into human milk, is also partially inactivated by milk itself (15). This is thought to be beneficial for very preterm infants as they are at the greatest risk for symptomatic infection via human milk (16, 17). However, this immune activity can be altered by pasteurization, given that certain antiviral components are negatively impacted by prolonged heating. Reduced antiviral activity was reported for CMV, herpes simplex virus 2, and respiratory syncytial virus after donor human milk underwent HoP (18). Given the negative impact of HoP on milk bioactive components in addition to heat-sensitive vitamins, there is considerable interest in exploring less harsh alternative pasteurization methods including high temperature short time (HTST), UV-C irradiation, ultrasonic processing, and high-pressure processing (HPP) (19).

High-pressure processing, a non-thermal method widely used in the food industry, better maintains bioactive components of human milk compared to HoP; however, its effect on the innate antiviral activity of human milk is unknown (20). Thus, the primary objective of this study was to investigate the impact of HoP and HPP processing on the anti-CMV activity in human milk. As human milk components are known to target the lipid membrane of enveloped viruses (21), the antiviral activity against hepatitis A virus (HAV) was tested as a proxy for non-enveloped viruses, which may also be present in human milk. Though not commonly present in human milk, HAV is often used to test processing procedures in the food industry (22). To our knowledge, no study has directly compared the inactivation of CMV and HAV by human milk processed by HoP and HPP. Since HPP preserves heat-sensitive immune components known to contribute to human milk’s antiviral activity, we hypothesized that milk treated by HPP would inactivate inoculated virus to a greater extent than milk treated by HoP.

## Materials and Methods

**FIGURE 1:**
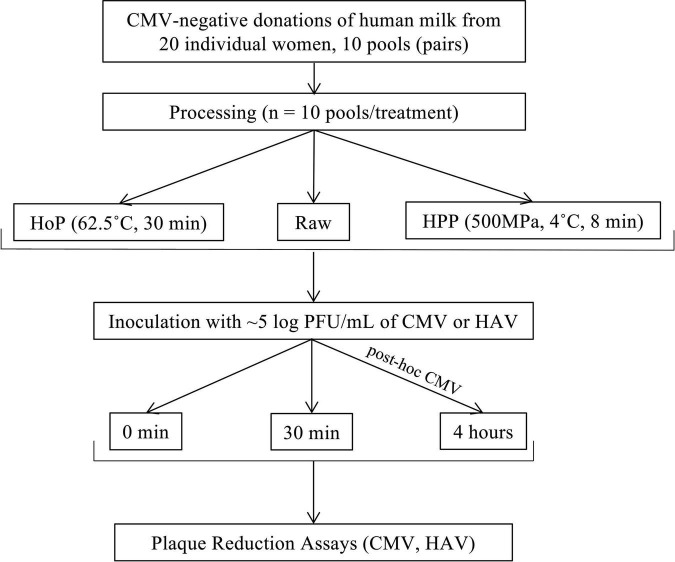
Study flow diagram. The flow chart demonstrates sample collection, processing, and experimental design. HoP, Holder pasteurization; HPP, high-pressure processing.

The experimental design used to assess the antiviral activity of raw and pasteurized donor milk is summarized in Figure 1. This study was approved by research ethics boards at The Hospital for Sick Children and Sinai Health, Toronto, ON, Canada.

### Human Milk Collection and Processing

Informed consent for the use of milk for research purposes is routinely obtained from each donor to the Rogers Hixon Ontario Human Milk Bank. CMV-negative donations of human milk [screened by polymerase chain reaction (Roche Cobas 6800)] from 20 women were collected, thawed overnight at 4°C, and pooled into one of 10 glass flasks to mimic milk bank practices. Each pool was composed of milk from two different women. Pools were then gently warmed to 37°C before being divided into three aliquots. One aliquot remained untreated (raw) and one each underwent HoP and HPP, respectively. All milk was then frozen immediately and stored at −80°C. The median (interquartile range) days postpartum that milk was expressed for each of the 10 donor human milk pools was 66 (28–105) days. Prior to pooling and subsequent processing, milk was frozen at −20°C on average for 124 days (119–140), within the recommended guidelines (23).

Each pool of milk to undergo HoP or HPP was first thawed overnight (4°C). Pools to remain raw were kept frozen at −80°C until antiviral testing. HoP was conducted using a shaker water bath, equipped with a temperature probe and data logger (Supplementary Figure 1), to mimic milk bank practices in the laboratory (23). Pools were heated to 62.5°C, held for 30 min, and then immediately cooled to 4°C in an ice water bath. HPP was conducted using an industrial-grade pressurizer (Model 135, Hiperbaric) using water (500 MPa, 8 min, 4°C) as the transmission medium (Université Laval, Quebec City, QC, Canada). Prior to sampling, all pools were mixed by gentle inversion. Samples were then collected and immediately placed at −80°C until antiviral testing.

### Assessment of Antiviral Activity

Viral suspensions and quantification by plaque reduction assay were done according to Pitino et al. (24). CMV-AD169 and HAV strain HM175/18f obtained from American Type Culture Collection (Manassas, VA, United States) were used to produce high titer suspensions following previously published protocols (25–27).

All experiments to assess antiviral activity were conducted in duplicate. Briefly, raw, HoP, and HPP samples (*n* = 10 per treatment) were thawed and gently vortexed. For each experiment, 900 μL of human milk was spiked with either 100 μL of previously titered CMV suspension [3.2 × 10^6^ plaque-forming units (PFU)/mL] or HAV suspension (5.1 × 10^6^ PFU/mL) to attain a final concentration of ∼5-log PFU/mL in milk. As a positive control, 900 μL of Minimum Essential Medium (Gibco) and Dulbecco’s Modified Eagle Medium (Gibco) were similarly inoculated with 100 μL CMV and HAV, respectively. Once inoculated, spiked samples of human milk or cell culture media were immediately assayed. Concurrently, spiked samples were incubated at room temperature (∼22°C) for 30 min, previously shown to inactivate inoculated SARS-CoV-2 in human milk by 1-log on average (range of 0–2-log) (14). Post-incubation, CMV and HAV in milk and cell culture media were measured as described above, after sample filtration (0.45 μm polyvinylidene fluoride) to limit the potential for bacterial contamination. To determine whether increasing incubation time affected antiviral activity, *post hoc* experiments using a 4 h incubation time were conducted only if initial reductions of CMV or HAV were detected at 30 min. Plaque reduction assays were conducted in duplicate for each replicate of the experiment. The limit of detection (LOD) for the plaque reduction assays of milk were 400 and 50 PFU/mL for CMV and HAV, respectively based on the volume plated (0.25 mL for CMV and 2.0 mL for HAV) and minimum dilution (1:100) required to overcome cytotoxicity (14). The LOD in cell culture media controls was 100-fold lower than milk as samples were assayed undiluted (4 PFU/mL CMV and 0.5 PFU/mL HAV).

### Statistical Analyses

Means of the outcome variables (concentrations of CMV and HAV) were compared among pasteurization groups (raw, HoP, and HPP) using linear mixed-effects models treating subject as a random effect. The models tested an incubation time by pasteurization group interaction. Non-significant interaction terms were removed, and the model was re-run. *Post hoc* pairwise comparisons between groups were reported. If data were non-normally distributed, a non-parametric repeated-measures rank-based analysis was carried out. For statistical purposes only, values below the LOD were assigned a value of half the LOD. *P*-values < 0.05 were considered statistically significant. All analyses were run using SAS Statistical software (version 9.4; SAS Institute, Cary, NC, United States), and data visualization was conducted using R Statistical Software (R Foundation for Statistical Computing version 4.1.2).

## Results

### Inactivation of Cytomegalovirus and Hepatitis A Virus in Donor Human Milk Pools

**FIGURE 2:**
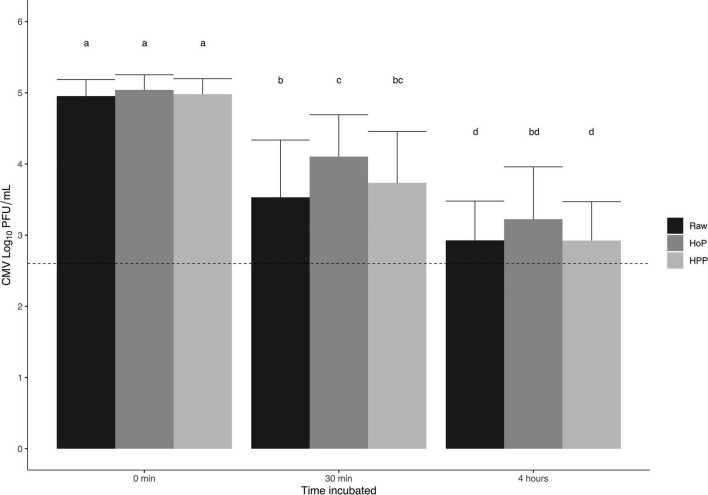
Recovered titers of CMV from inoculated milk following incubation at room temperature. Mean CMV log PFU/mL for raw, Holder pasteurization (HoP), and high-pressure processing (HPP) treated pools. Each bar represents the mean of the pools plated in duplicate after 0 min, 30 min, and 4 h incubations (room temperature, ∼22°C) with error bars representing the standard deviation; dashed line represents the limit of detection. Statistical analyses were conducted using linear mixed effect models with *post hoc* pairwise comparisons. Different letters denote statistical significance (*P* < 0.05).

Concentrations of CMV and HAV are presented as mean log-transformed values (standard deviation) where appropriate. Each of the 10 inoculated pools yielded a final concentration of approximately 5.5- and 5.7-log PFU/mL for CMV and HAV, respectively. Titration of CMV yielded an average concentration of 5.0 (0.2) log PFU/mL at 0 min and was 3.4 (0.8), 4.1 (0.6), and 3.7 (0.7) log PFU/mL in raw, HoP, and HPP milk pools respectively after 30 min (Figure 2). There were no differences among treatments at 0 min. At 30 min, the concentration of CMV for all treatments was significantly lower compared to 0 min (all *P* < 0.0001); however, the concentration in raw milk (1.4-log PFU reduction vs. 0 min) was significantly lower than HoP milk (0.9-log PFU reduction vs. 0 min) (*P* = 0.0069). CMV added to HPP milk was not significantly different compared to raw or HoP.

**FIGURE 3:**
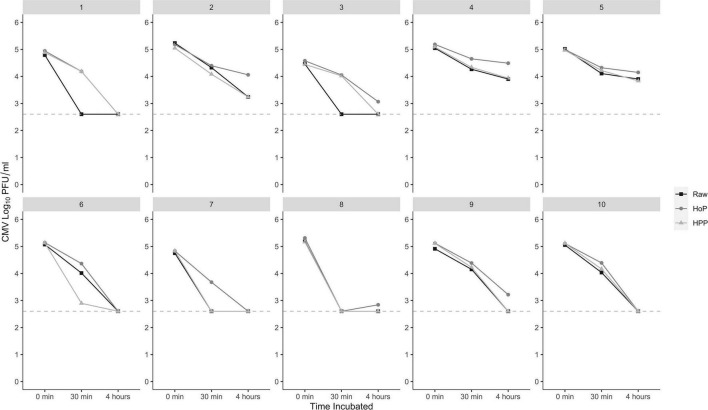
Distribution of anti-CMV activity per pool up to 4 h. Individual donor human milk pools (panels) exhibit varying degrees of reduction in detectable CMV after 30 min and 4 h incubations. Plaque reduction assays were plated in duplicate; the dashed line represents the limit of detection. HoP, Holder pasteurization; HPP, high-pressure processing.

Results from *post hoc* experiments where CMV was incubated in milk for 4 h demonstrated an additional 0.6 (0.2), 0.9 (0.1), and 0.8 (0.2) log PFU/mL following raw, HoP, and HPP, respectively (Figure 2) compared to reductions at 30 min. No treatments were significantly different from each other after 4 h. The concentrations of CMV at 4 h in raw and HPP samples were significantly lower than all treatments at 30 min (all *P* < 0.011), while HoP samples were only significantly different from HoP treated pools at 30 min (*P* = 0.0008). Recovered titers of CMV in individual donor human milk pools are presented in Figure 3. The concentration of CMV in inoculated cell culture medium was not affected by incubation and was measured at 5.6 (0.1) log PFU/mL at all timepoints.

Initial concentrations of HAV at 0 min were 5.7 (0.2), 5.8 (0.1), and 5.8 (0.1) log PFU/mL for raw, HoP, and HPP samples, respectively. There were no significant differences after 30 min (5.6-log PFU/mL) among or between treatments (Supplementary Figure 2). The concentration of HAV in inoculated cell culture media was not affected by incubation and was 5.6-log PFU/mL at both 0 and 30 min.

## Discussion

Although the antiviral activity of milk has been well documented, this is the first study to our knowledge directly comparing the antiviral activity in donated human milk treated by HoP and HPP against a representative enveloped and non-enveloped virus (15, 18). Both raw and pasteurized (HoP or HPP) human milk exhibited anti-CMV activity, with CMV titers declining over 4 h of incubation at room temperature; the maximum recommended time to store human milk at room temperature (23). In contrast, no anti-HAV activity was detectable after 30 min of incubation. As previously shown, anti-CMV activity was significantly higher in raw milk compared to milk treated by HoP after 30 min (15). In our study, HPP-treated milk inactivated CMV to a degree that appeared to be intermediate between raw milk and HoP milk at 30 min. Similarly, Keulen et al. recently reported HPP treated milk had greater antiviral activity against SARS-CoV-2 than HoP (28). We speculate that some of the differences between raw milk and HoP milk are due to the preservation of heat-sensitive components that have antiviral activity.

Treatment of milk by HPP (500 MPa, 8 min, 4°C), the parameters in this study, has been previously shown to better preserve bile salt stimulated lipase, lactoferrin, and lysozyme, in comparison to alternative methods including HoP, flash heating, and UV-C irradiation (20, 29). Although other parameters have been explored, recent evidence has suggested 500 MPa (8 or 10 min) maximizes the preservation of these bioactive components while ensuring microbiological safety [<1 colony forming unit (CFU)/mL] (24). Despite these benefits, it is important to note that HPP is associated with a high start-up cost, highlighting the need to explore more affordable and smaller-scale equipment for use in milk bank settings. Other alternative methods such as HTST should also be investigated in comparison to HPP, given that antiviral activity (CMV, herpes simplex virus 2, and respiratory syncytial virus) and some bioactive components are better retained in HTST milk versus HoP (18, 30).

The fact that the CMV antiviral activity persisted in all raw and treated milk suggests that reductions in detectable CMV are mediated by physical or biochemical properties of the milk which are minimally affected by heating or isostatic pressurization. However, several other mechanisms may affect antiviral activity. Oligosaccharides and incomplete inactivation of some antiviral proteins (lactoferrin and lysozyme) may provide antiviral activity (9). Since the fatty acid composition of milk is generally unaltered by processing, non-enzymatic lipolysis of milk triglycerides could also have released fatty acids (31, 32). Mechanistically, certain free fatty acids in milk exert antiviral activity via the disruption of the viral envelope, which could explain why inactivation of CMV, an enveloped virus, was observed, while no reductions were seen for HAV, a non-enveloped virus (33). Finally, increased acidity as a by-product of lipolysis could also independently contribute to antiviral activity (34). Notably, we observed high variability in the anti-CMV activity across the 10 pools of milk, whereby the mean difference of CMV log reduction ranged from 0.5 to 2 (Figure 3). In certain pools, inactivation of CMV was rapid after 30 min yielding a reduction below the LOD in all treatments; in others, inactivation appeared to be more gradual up to 4 h (Figure 3). While the LOD for the plaque reduction assay may have limited our ability to detect larger changes, no significant reductions were observed in the positive controls using cell culture media at any timepoint.

As serology screening for antibodies is not standard practice for North American milk banks, this was not accounted for in our pragmatic study; however, given the high prevalence of CMV in the population (35), it is likely that some of the milk could have contained antibodies to CMV in response to a previous maternal infection without detectable viral DNA. Since human milk contains antibodies that are partially retained after processing, we cannot discount the possibility that pools of milk may have functional antibodies to CMV (6, 28, 29). Antiviral activity against CMV was shown to remain prevalent after depletion of secretory IgA, in both IgG positive and negative mothers, suggesting components in milk other than antibodies may be also contributing to the antiviral activity (15).

A strength of this study is our paired design, which permitted comparisons among all treatment groups. Despite high variability between the pools, anti-CMV activity was maintained up to 4 h regardless of treatment. Our study has several limitations. Our sample size meant that we could not evaluate the impact of the lactational stage of milk on antiviral activity. Furthermore, our experiments were conducted on pools consisting primarily of mature milk, and thus, our results could have differed if they were conducted on early milk given differences in the concentration of bioactive components. Previous reports suggest that greater anti-CMV activity is associated with earlier lactational stages (15). Our ability to detect differences between treatments may have been greater if milk with innately greater anti-CMV activity was used. Although not assessed in this pragmatic study, a potential limitation is that the concentration of bioactive components may also be impacted by certain donor characteristics; however, pooling milk from multiple donors would have likely minimized any potential impact of these characteristics. We only used two representative viruses and further research is warranted to better understand whether processing, either by HoP or HPP, affects the antiviral activity of milk against other viruses which can be present in human milk. There is currently no consensus in the literature to the degree with which human milk inactivates other enveloped viruses, including human immunodeficiency virus, herpes simplex viruses, and respiratory syncytial virus (18, 36–38). Although we observed no anti-HAV activity, it is possible that other non-enveloped viruses could be partially inactivated by human milk. In fact, previous studies have demonstrated that human milk can partially inactivate rotavirus, a non-enveloped virus; further evidence is needed to examine other non-enveloped viruses (39, 40).

Given concerns over the negative impact of HoP on milk composition, including destruction of heat-sensitive vitamins and some bioactive components, HPP was explored as a promising, non-thermal alternative for milk bank processing. We observed that HPP treated milk maintains anti-CMV activity comparable to raw milk and HoP milk for up to 4 h. Further research is needed to understand the comparability of HoP and HPP in retaining the antiviral activity of other viruses common in human milk or to which a vulnerable infant may be exposed.

## Data Availability Statement

The raw data supporting the conclusions of this article will be made available by the authors, without undue reservation.

## Ethics Statement

The studies involving human participants were reviewed and approved by the research ethics boards at The Hospital for Sick Children and Sinai Health, Toronto, ON, Canada. The patients/participants provided their written informed consent to participate in this study.

## Author Contributions

AK, MP, SU, and DO contributed to the conception and design of the study. AK, MP, and VP carried out the initial experiments and performed statistical analyses. AK wrote the initial draft of the manuscript. All authors contributed to manuscript revision, read, and approved the submitted version.

## Conflict of Interest

The authors declare that the research was conducted in the absence of any commercial or financial relationships that could be construed as a potential conflict of interest.

## Publisher’s Note

All claims expressed in this article are solely those of the authors and do not necessarily represent those of their affiliated organizations, or those of the publisher, the editors and the reviewers. Any product that may be evaluated in this article, or claim that may be made by its manufacturer, is not guaranteed or endorsed by the publisher.

## Abbreviations

CMV: cytomegalovirus
HAV: hepatitis A virus
HoP: Holder pasteurization
HPP: high-pressure processing
HTST: high temperature short time
Ig: immunoglobulin
LOD: limit of detection
PFU: plaque-forming unit
SARS-CoV-2: severe acute respiratory syndrome coronavirus-2.
