# Supplementary material for: Preservation of Anti-cytomegalovirus Activity in Human Milk Following High-Pressure Processing Compared to Holder Pasteurization
Source: Front Nutr. 2022 May 19;9:918814. doi: 10.3389/fnut.2022.918814 (PMC9160983; doi:10.3389/fnut.2022.918814)
Supplement: Supplementary file 1 [file Image_1.PDF]

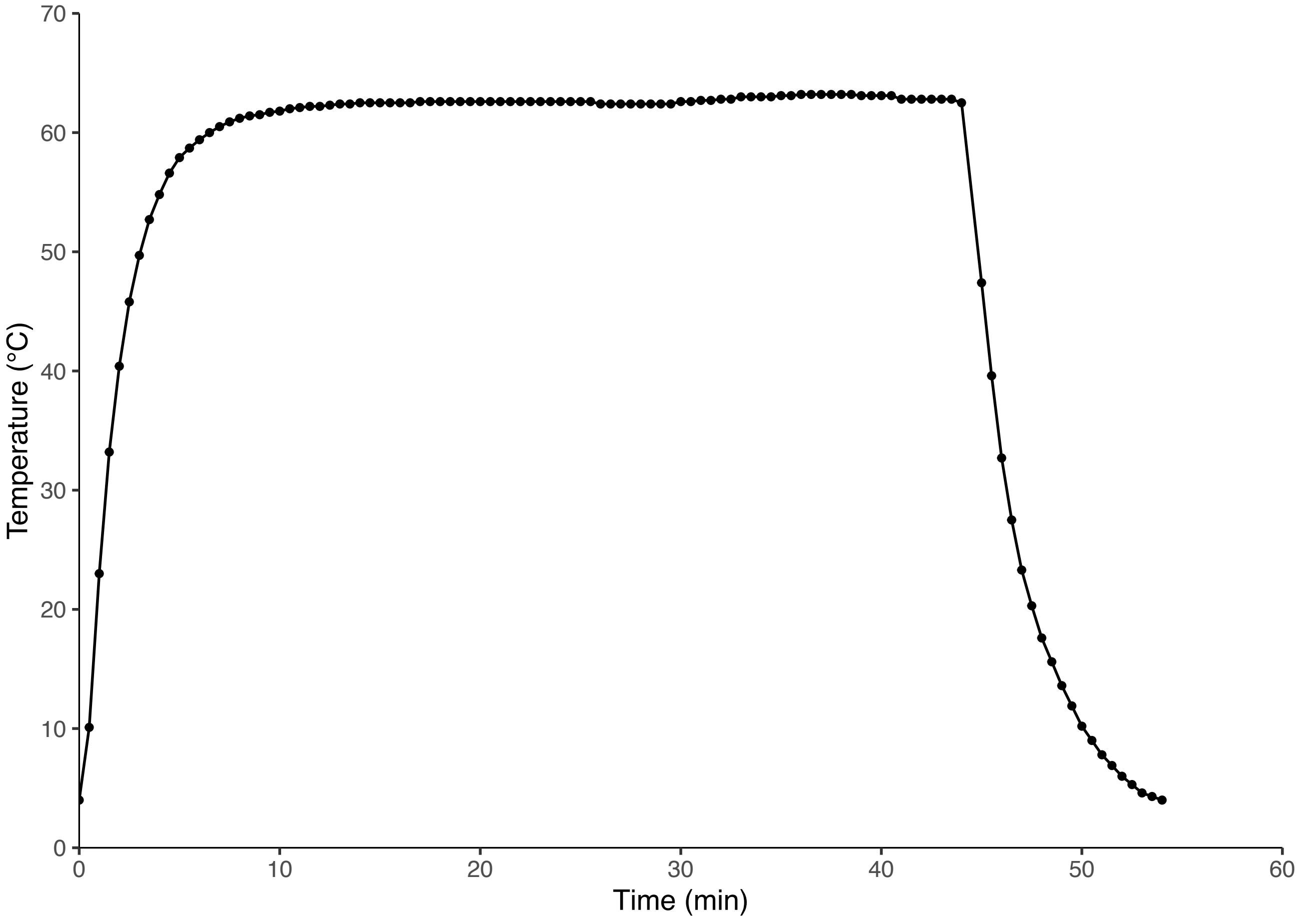

Supplemental Figure 1. Holder pasteurization of donor milk pools.

Pools were thawed overnight to 4°C and subsequently submerged in a shaker water bath until reaching 62.5°C. This temperature was attained at 14 min and maintained for 30 min (Holder pasteurization). Pools were then rapidly cooled to 4°C in an ice water bath.
