# Supplementary material for: Preservation of Anti-cytomegalovirus Activity in Human Milk Following High-Pressure Processing Compared to Holder Pasteurization
Source: Front Nutr. 2022 May 19;9:918814. doi: 10.3389/fnut.2022.918814 (PMC9160983; doi:10.3389/fnut.2022.918814)
Supplement: Supplementary file 2 [file Image_2.PDF]

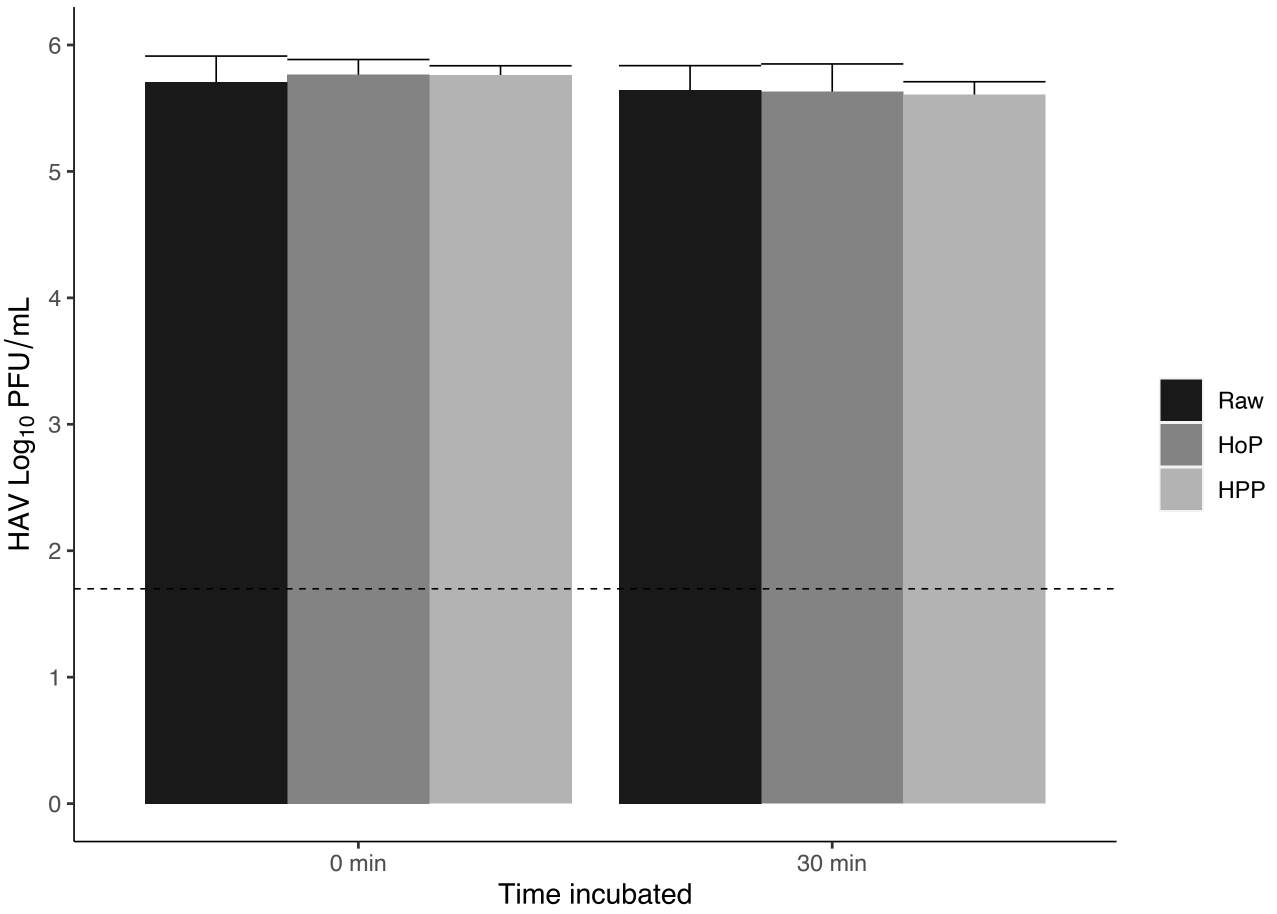

Supplemental Figure 2. Recovered titers of HAV from inoculated milk following room temperature incubation.

Mean HAV log PFU/mL for raw, Holder pasteurization (HoP), and high pressure processing (HPP) treated pools. Each bar represents the mean of the pools plated in duplicate after 0 and 30 min incubations with error bars representing the standard deviation; dashed line represents limit of detection. Statistical analyses were conducted using linear mixed effect models ($P < 0.05$). No statistically significant differences were observed.
